# Transition steps in peroxide reduction and a molecular switch for peroxide robustness of prokaryotic peroxiredoxins

**DOI:** 10.1038/srep37610

**Published:** 2016-11-28

**Authors:** Neelagandan Kamariah, Mun Foong Sek, Birgit Eisenhaber, Frank Eisenhaber, Gerhard Grüber

**Affiliations:** 1 Bioinformatics Institute, Agency for Science, Technology and Research (A*STAR), 30 Biopolis Street, #07-01 Matrix, Singapore 138671, Republic of Singapore; 2 School of Biological Sciences, Nanyang Technological University, 60 Nanyang Drive, Singapore 637551, Republic of Singapore; 3 School of Computer Engineering, Nanyang Technological University (NTU), 50 Nanyang Drive, Singapore 637553, Republic of Singapore

## Abstract

In addition to their antioxidant function, the eukaryotic peroxiredoxins (Prxs) facilitate peroxide-mediated signaling by undergoing controlled inactivation by peroxide-driven over-oxidation. In general, the bacterial enzyme lacks this controlled inactivation mechanism, making it more resistant to high H_2_O_2_ concentrations. During peroxide reduction, the active site alternates between reduced, fully folded (FF), and oxidized, locally unfolded (LU) conformations. Here we present novel insights into the divergence of bacterial and human Prxs in robustness and sensitivity to inactivation, respectively. Structural details provide new insights into sub-steps during the catalysis of peroxide reduction, enabling the transition from an FF to a LU conformation. Complementary to mutational and enzymatic results, these data unravel the essential role of the C-terminal tail of bacterial Prxs to act as a molecular switch, mediating the transition from an FF to a LU state. In addition, we propose that the C-terminal tail has influence on the propensity of the disulphide bond formation, indicating that as a consequence on the robustness and sensitivity to over-oxidation. Finally, a physical linkage between the catalytic site, the C-terminal tail and the oligomer interface is described.

Reactive oxygen species (ROS) are inevitable byproducts of normal aerobic metabolism, which at high levels can inflict damages on DNA, lipids and proteins. The intracellular concentration of peroxides is tightly maintained at very low levels by the oxidant-specific sensors that regulates the expression of oxidant-scavenger enzymes^1^,^2^. Peroxiredoxins (Prxs), a class of thiol-based peroxidases, present in all biological kingdoms from bacteria to human and consisting of six evolutionary subfamilies (Prx1, Prx5, Prx6, Tpx, PrxQ, and AhpE), are the dominant enzymes responsible for the reduction of over 90% of mitochondrial and cytoplasmic H_2_O_2_^3^,^4^. These enzymes have recently grabbed attention not only due to their antioxidant function, but also for their additional significance in a broad range of cellular events including modulation of local intracellular H_2_O_2_ level required for signaling events^5^, and peroxide sensing for activation of transcription factors that control the expression of antioxidant enzymes^6^,^7^. Besides the importance of Prxs in keeping thioredoxins in a reduced state for maintaining cell viability under oxidative stress^8^, they act as peroxinitrite reductases^9^, chaperone holdases^10^, protein foldases^11^, and regulators of circadian rhythm^12^. Because of their fundamental cellular functions, Prxs are implicated in aging, cancer, cardiovascular diseases, diabetes and neurodegeneration, and are therefore appealing as therapeutic targets^13^,^14^.

The Prx subfamily enzyme Prx1, also typically called 2-Cys Prx, is among the most highly expressed, soluble proteins in the cell^15^, and is characterized by two conserved cysteine residues. One is represented by the peroxidatic cysteine (C_P_), which becomes selectively oxidized by H_2_O_2_ to the C_P_–SOH intermediate. This C_P_ further reacts with the so-called resolving cysteine (C_R_). The C_R_ is located at the C-terminus of the other subunit of the basic functional dimer, forming an intermolecular disulphide with the C_P_. Regeneration of the intermolecular disulphide bridge occurs via the Peroxiredoxin reductases (PrxR), enabling a continuous catalytic cycle^16^,^17^.

As shown in Fig. 1, the detailed structural analysis of bacterial Prx1, called Alkyl hydroperoxide reductase subunit C (AhpC), reveals two distinct active site conformations linked with their catalytic cycle. In the reduced state, the active site is competent for productive substrate binding and is in a so-called fully folded (FF) conformation, where the peroxidatic cysteine, C_P_47 (according to the *Escherichia coli* subunit AhpC numbering), is part of the α2-helix and is located at the bottom of the catalytic cavity, formed by the conserved residues, P40, T44 and R120. In parallel, the resolving cysteine (C_R_166), placed at the C-terminal tail of an adjacent subunit, is folded across the active site, placing C_P_ and C_R_ 14 Å away and in opposite orientation^18^ (Fig. 1). In the oxidized state, the C_P_ and C_R_ are disulphide bonded in the so-called locally unfolded (LU) conformation, where (*i*) C_P_ of helix α2 is partially unwound, orienting C_P_ towards C_R_, and (*ii*) the C-terminal tail unfolds from the active site region and becomes disordered^19^,^20^ (Fig. 1). Additionally, the redox-state dependent active site conformations are proposed to modulate the quaternary structure of Prxs, whereas the reduced FF active site favors the decamer formation and the oxidized LU active site weakens the decamer into a dimer^19^. Our recent studies on the *Escherichia coli* AhpC (*Ec*AhpC) explored the different functional role of its C-terminal tail, as influenced by the redox-state of the enzyme. In the oxidized state, the C-terminal tail of *Ec*AhpC, which includes amino acids 172 to 187, is essential for regeneration by its specific Alkyl hydroperoxide reductase subunit F (AhpF) (Fig. 1). At the same time, the C-terminal tail is essential for the formation of a stable doughnut-shaped decamer under reduced conditions^21^,^22^,^23^.

Based on the elucidation of distinct redox-state-linked active-site conformations, it has been proposed that during the formation of the C_P_-SOH intermediate, the active site should move out of the FF conformation to facilitate the formation of the disulphide bond with C_R_^16^. Nevertheless, a detailed picture of the structural transition(s) between the FF and LU state during the catalysis is missing. In contrast, the structural studies on eukaryotic Prxs revealed that the FF active site conformation persists during and after oxidation of C_P_ to C_P_-SOH (Fig. 1), which eventually promotes the over-oxidation of C_P_-SOH into C_P_-SO_2_H/C_P_-SO_3_H, and finally inactivates the enzyme^24^,^25^. The presence of two conserved sequence features in human and other eukaryotic Prxs, the so-called GGLG motif and the extended C-terminal helix containing the YF motif, are thought to be essential for regulating H_2_O_2_ mediated signal transduction^5^,^18^. However, the greater importance of the C-terminal helix for sensitivity to over-oxidation has been confirmed^25^,^26^. The C-terminal helix with its YF motif folds across the active site, thereby delaying the conformational change from FF to LU, and favoring over-oxidation^15^ (Fig. 1). It has been implicated that the disulphide formation or over-oxidation depends on the rate of FF to LU transitions. According to the proposed floodgate model, over-oxidation of Prxs facilitates the local accumulation of intracellular H_2_O_2_ required for signaling events^18^. Finally, the enzyme sulfiredoxin (Srx) catalyzes the repair of over-oxidized Prxs, to restore their peroxidase activity^27^.

In order to (*i*) gain deep insight into the delicate balance between the FF and LU active site during the catalytic cycle, (*ii*) to explore the underpinning role of the C-terminal tail in active site conformation, and (*iii*) to understand the features making the bacterial and human Prxs more robust and sensitive to inactivation, respectively, a combination of genetic engineering, enzyme kinetics and crystallography were used to establish the values of kinetic parameters and sensitivity for inactivation using the well-established *E. coli* AhpC and its mutants. By generating and determining the structure of a chimeric *Ec*AhpC_1-186_-YFSKHN, which includes the extended C-terminal helix with the YF motif of human Prx2, we deduced for the first time the intermediate active-site conformations, lying between the FF and LU conformations. Furthermore, the studies reveal that the C-terminal tail acts as a molecular switch to mediate the structural transition between the FF and LU state. Finally, we propose the detailed conformational transition states that accompany the peroxide reduction and over-oxidation cycle, providing novel insight into the evolutionary divergence of the resistant and sensitive Prxs catalysts of bacteria and human, respectively.

## Results

### Sensitivity to oxidative inactivation of a novel chimeric *Ec*AhpC_1-186_-YFSKHN protein

The bacterial Prx is less sensitive to inactivation by hyper-oxidation, whereas the human Prx is sensitive towards inactivation with its C-terminal helix (YFSKHN-helix), including the conserved residues YF, playing a vital role^18^. The question arises, whether the apparent sensitivity to over-oxidation exerted by the C-terminal helix of human Prx can be transformed into Prxs of prokaryotes. We used the mechanistically well understood *E. coli* as a prototype to construct a chimeric Prx, *Ec*AhpC_1-186_-YFSKHN, composed of *Ec*AhpC (residues 1–186) and the C-terminal YFSKHN-segment of human Prx 2, to be compared with wild-type (WT) *Ec*AhpC (Fig. 2A). The *E. coli* TrxR-Trx system, the common reductase system to characterize Prxs, was used in the peroxidase assay, in which NADPH-oxidation by TrxR provides the electrons via Trx to *Ec*AhpC for H_2_O_2_ reduction.

As shown in Fig. 2A, WT *Ec*AhpC reacts with 1 mM H_2_O_2_ and actively consumes NAPDH. Increasing the H_2_O_2_ concentration to 10 and 20 mM caused an activity drop of ~12% and ~18%, respectively. The inhibitory effect of H_2_O_2_ to *Ec*AhpC increased significantly at a concentration of 30 mM, resulting in an enzyme activity reduction of ~44%. In comparison, an increase of 10 mM H_2_O_2_ decreased the chimeric *Ec*AhpC_1-186_-YFSKHN enzyme activity by already ~33% (Fig. 2B,C), followed by a further drastic decrease of ~50% in the presence of 20 mM H_2_O_2_, and finally to a ~64% drop with 30 mM H_2_O_2_ (Fig. 2C). The data indicate that WT *Ec*AhpC is resistant to inactivation up to 20 mM H_2_O_2_, as it shows little variation for peroxidase activity. In contrast, the peroxidase activity of chimeric *Ec*AhpC_1-186_-YFSKHN is affected at H_2_O_2_ concentrations above 1 mM, revealing that the transformation of the human C-terminal YFSKHN-helix inside the bacterial *Ec*AhpC increased its sensitivity to over-oxidation.

### Effect of the human YFSKHN-segment on catalysis

In order to address the question, whether the YFSKHN-helix affects the peroxidase activity, the catalytic turnover number (*k*_*cat*_), and the Michaelis-constant (*K*_*m*_) for substrate-binding were determined. The peroxide-dependent NAPDH-oxidation of *Ec*AhpC and *Ec*AhpC_1-186_-YFSKHN were measured with varying concentrations of H_2_O_2_ and Fig. 2D shows the NAPDH-oxidation at 30 μM H_2_O_2_ as a representative of all other measurements done. WT *Ec*AhpC and *Ec*AhpC_1-186_-YFSKHN exhibited typical substrate saturation kinetics of the Michaelis-Menten type (Fig. 2E,F). The determined kinetic constants and catalytic efficiency are reported in Table 1. The *Km*- and *k*_*cat*_-value of *Ec*AhpC were determined to be 1.44 μM and 0.09 s^−1^, respectively. In case of *Ec*AhpC_1-186_-YFSKHN a higher *Km* of 1.77 μM and slightly lower *k*_*cat*_ value of 0.082 s^−1^ was calculated, resulting in a lower catalytic efficiency of *Ec*AhpC_1-186_-YFSKHN [*k*_*cat*_*/K*_*m*_ (H_2_O_2_)], 4.8 × 10^4^ M^−1^ s^−1^ when compared to the wild-type enzyme (6.2 × 10^4^ M^−1^ s^−1^).

### Crystallographic structure of oxidized and decameric-shaped *Ec*AhpC_1-186_-YFSKHN

To understand the structural effect of the human YFSKHN-helix in general, in particular inside the chimeric *Ec*AhpC_1-186_-YFSKHN, as well as to identify the individual amino acids essential for sensitivity to oxidative inactivation of Prxs, the crystal structure of oxidized *Ec*AhpC_1-186_-YFSKHN has been solved at 2.7 Å resolution. The asymmetric unit contains one decamer composed of five catalytic dimers (α_2_)_5_ with each subunit containing one catalytic active peroxidatic and one resolving cysteine (Fig. 3A,B). The subunit consists of a central seven-stranded β-sheet, flanked at one side by four and at the other side by two α-helices (Fig. 3C). Each subunit has a dimer and oligomer interface. The dimer interface is mainly stabilized by salt bridge and hydrogen bond interactions, while the oligomer interface is mainly stabilized by hydrophobic interactions. Superposition of the recently resolved oxidized *Ec*AhpC structure^20^ with the corresponding dimer of the oxidized *Ec*AhpC_1-186_-YFSKHN one resulted in an r.m.s.d. value of 0.4 Å. The intermolecular disulphide bond observed between C_P_47 and C_R_166′ in the dimer interface (Fig. 3B,C) confirms the oxidized state of the *Ec*AhpC_1-186_-YFSKHN structure. The peroxidatic cysteine (C_P_47), located in the first turn of helix α2, adopts an LU conformation in the oxidized structure (Fig. 3B,C and Supplementary Fig. S1A). In most of the disulphide bonded active sites of the decameric and oxidized *Ec*AhpC_1-186_-YFSKHN, the C-terminal arm beyond the resolving cysteine (C_R_166) is disordered, indicating the high flexibility of the C-terminus.

However, the structure in chain (H) could be resolved until residue 177, showing amino acids 167 to 177 oriented away from the active site and stacked against the neighboring symmetry molecule. Similarly, the C-termini of chains A, G and J are also stabilized by the packing interactions with the symmetry related molecules (Supplementary Fig. S1B,C). The average main chain B-factors analysis revealed three dynamic regions of oxidized *Ec*AhpC_1-186_-YFSKHN: (*i*) the C_P_47 containing α2-helix, (*ii*) helix α5 that contains residues S86 and T88, and (*iii*) the C-terminal tail region (Fig. 3D). The sulfate ions observed in the interface region of the structure, emphasis their role in stabilizing the oxidized AhpC in a decameric form in solution^23^.

### The active site conformation in reduced *Ec*AhpC_1-186_-YFSKHN

To shine a light on the redox modulated structural alterations in the active site, the crystallographic structure of reduced *Ec*AhpC_1-186_-YFSKHN was determined to 3.1 Å resolution (Fig. 4A). Except for the C-terminus, clear electron density was observed for all the residues and the resolving cysteine (chains C, E, F, H and J), which is located about 10 Å away from the peroxidatic one (Fig. 4B). No disulphide bond between C_P_47 and C_R_166′ was observed in any of the active site interfaces of the decameric *Ec*AhpC_1-186_-YFSKHN, confirming that the structure fairly represents the reduced form of *Ec*AhpC_1-186_-YFSKHN. In the reduced state, the typical FF active site is arranged in a way that the C_P_47 positions in the first turn of helix α2 and C_R_166′, containing the C-terminal arm, is folded across the dimer interface to come in proximity to the first turn of helix α2 of another subunit. This conformational alteration brings the C_P_ and C_R_’ to face opposite directions and to become separated by about 10 Å.

Interestingly, two different active site conformations were observed in the reduced *Ec*AhpC_1-186_-YFSKHN structure (Supplementary Fig. S2A), where the residues in the active site region are clearly defined in the electron density map except for the side chains of F45 and V46 (Fig. 4C,D). The first conformation (chains B-J) reveals that the peroxidatic C_P_47 adopts helical conformation, bringing the C_P_47 in a narrow solvent accessible pocket, which is surrounded by the highly conserved active site residues P40, T44 and R120 (Fig. 5A). In the second active site conformation (chain A), the first turn of α2-helix is partially unfolded to place C_P_47 in the loop conformation (Fig. 5A). However, no significant influence of crystal packing on the conformations of C_P_47 was observed in the reduced *Ec*AhpC_1-186_-YFSKHN structure (Supplementary Fig. S2B,C). A comparison of these two reduced active sites with the oxidized LU state of *Ec*AhpC_1-186_-YFSKHN reflects a significant difference in the loop region (T44-P48) (Fig. 5B), where the FF active site residues of the first active site of reduced *Ec*AhpC_1-186_-YFSKHN are rotated, to expose C_P_47 towards the C_R_166′, and finally forming a disulphide bond in the oxidized structure. While in the second active site of reduced *Ec*AhpC_1-186_-YFSKHN, the unfolded C_P_47 adopts a lower magnitude of rotation, thereby preventing its full exposure towards C_R_166′ to form a disulphide (Fig. 5B).

### FF to LU transition state like active site conformation

To gain insight into (*i*) the structural transition pathway between the FF and LU state, and (*ii*) the role of the C-terminal tail on the active site conformation, the two presented distinct active sites of reduced *Ec*AhpC_1-186_-YFSKHN were compared with a typical reduced FF and oxidized LU conformation, represented by the crystallographic structure of the *Salmonella typhimurium* AhpC^28^ (PDB ID: 4MA9) and *Ec*AhpC_1-186_-YFSKHN (see above), respectively. For clarity, we termed the first and second active sites of the reduced *Ec*AhpC_1-186_-YFSKHN as FF_like_ and LU_like_, respectively, to denote the folded and unfolded C_P_47 in the active site environments (Fig. 6A).

Firstly, the reduced *Ec*AhpC_1-186_-YFSKHN and the *S. typhimurium* AhpC structure (PDB ID: 4MA9) share an overall similarity indicated by an r.m.s.d. of 0.5 Å for the superposition of the catalytic dimers. When zoomed into the catalytic relevant α2-helix, the FF and FF_like_ active sites differ significantly (Fig. 6A). While the residues F45 and V46 are placed in the starting position of helix α2 inside the FF state, the α2-helix becomes unwound in the FF_like_ conformation and the main chain Cα atoms F45 and V46 shifted by about 0.9 Å. Similarly, in the FF_like_ conformation the main chain Cα atoms of C_P_47 and P48 moved about 0.6 Å and 0.5 Å, respectively, compared to their corresponding positions in the FF state (Fig. 6A). Furthermore, no obvious difference in the main chain conformations of the loop amino acids P40, T44 and R120 were observed between the two conformations.

The comparison of the FF (PDB ID: 4MA9) and LU_like_ active site (see above) shows, significant structural variation in the first turn of helix α2 and the loop region (Fig. 6B,C). Due to the partial unfolding of the α2-helix, the main chain Cα atoms of V44, F45, V46, C47 and P48 of the FF active site moved by 0.4, 1.8, 1.9, 5.1 and 1.1 Å, respectively, to achieve the LU_like_ state positions. As shown in Fig. 6B,C, the same residues moved during the transition from the FF to the LU state but with a higher magnitude of 1.4, 3.8, 3.8, 5.9 and 1.7 Å, respectively. Because of the magnitude of displacement, the data indicate that the FF_like_ and LU_like_ state, adopt intermediate conformations, lying between the catalytic FF and LU conformation. In addition, significant differences in the backbone torsion angles were determined between the FF, LU_like_ and LU active sites (Supplementary Table S1). Taken together, it can be proposed that the FF_like_ and LU_like_ active sites mimic the initial and the intermediate phase of the structural deformation that occurs during the redox-modulated FF to LU conformational switch.

### Oligomer interface region

The oligomeric behavior of Prxs is proposed to be redox modulated^29^. Under reduced conditions, the FF active site buttresses the oligomer interface, and favors decameric ring formation. Whereas under oxidized conditions, the LU active site mediates the restructuring of the oligomer interface region, resulting into lower order oligomers^19^. A structural view to the oligomeric interface in Fig. 7 highlights differences of the distinct active site loop conformations observed between FF, FF_like_, LU_like_ and LU, where the C_P_-containing loop residues of the LU conformation are pulled away from the oligomer interface. This facilitates the destabilization of the oligomer interface through structural rearrangements of F43 and F45, which participate in the interface region^19^,^29^. The rest of the interface region residues remain unchanged in the crystal structures.

### Effect of the C-terminal tail on substrate-binding and catalytic efficiency

By genetically engineering and enzymatically characterizing the *Ec*AhpC mutants AhpC_1–172_, AhpC_I187G_ and AhpC_S86A,T88A_, the questions were addressed, whether the folding of the C-terminal tail across the active site region as well as the dynamic region of helix α5 with residues S86 and T88 (Fig. 8A), influences the enzyme kinetics.

Like WT *Ec*AhpC, all *Ec*AhpC mutants exhibited Michaelis-Menten substrate saturation kinetics with a *k*_*cat*_ of 0.089 s^−1^ for *Ec*AhpC_I187G_ and *Ec*AhpC_S86A,T88A_, a value similar to *Ec*AhpC (Table 1, Fig. 8B–E). In comparison, the *k*_*cat*_ of 0.082 s^−1^ of the C-terminal truncated *Ec*AhpC_1–172_ was slightly lower but similar to the one determined for the chimeric *Ec*AhpC_1-186_-YFSKHN. In contrast to *Ec*AhpC_1-186_-YFSKHN and WT *Ec*AhpC, all three *Ec*AhpC mutants showed a significant decrease in H_2_O_2_-binding, with *Km* values of 8.26 μM, 9.74 μM, and 9.45 μM for *Ec*AhpC_I187G_, *Ec*AhpC_S86A,T88A_ and the C-terminal truncated *Ec*AhpC_1–172_, respectively. The increased *Km* values suggest that the active site binding pocket is impaired in all *Ec*AhpC mutants. The respective values of catalytic efficiency of the enzyme [*k*_*cat*_*/K*_*m*_ (H_2_O_2_)] determined for these mutants are 1.0 × 10^4^, 9.0 × 10^3^, and 8.6 × 10^3^ for *Ec*AhpC_I187G_, *Ec*AhpC_S86A,T88A_ and *Ec*AhpC_1–172_, respectively (Table 1).

### The role of the C-terminus of bacterial AhpC in H_2_O_2_ robustness

Revealing that the transformation of the human C-terminal YFSKHN-helix inside the bacterial *Ec*AhpC increased its sensitivity to over-oxidation, leads now to the question of whether the C-terminus of bacterial 2-Cys Prxs keeps the enzyme more robust and enabling the prokaryotes to survive under higher peroxide concentrations. To test this, the ability of the two C-terminal variants, *Ec*AhpC_1–172_ and *Ec*AhpC_I187G,_ to inactivation was studied. As shown in Fig. 9A,B, *Ec*AhpC_1–172_ and *Ec*AhpC_I187G_ showed NADPH consumption at 1 mM H_2_O_2_ concentration. Increase of H_2_O_2_ (10 mM) dropped the peroxidase activity of *Ec*AhpC_1–172_ and *Ec*AhpC_I187G_ by ~32% and ~34%, respectively (Figs 2C and 9A,B). The inhibitory effect of H_2_O_2_ in *Ec*AhpC_1–172_ and *Ec*AhpC_I187G_ increased significantly at a concentration of 30 mM, resulting in an enzyme reduction of ~64% and ~62%, respectively. These values are comparable to the once of the chimeric *Ec*AhpC_1-186_-YFSKHN, which were determined to be ~33% at a concentration of 10 mM H_2_O_2_ followed by a further drastic decrease of 50% in the presence of 20 mM H_2_O_2_, and finally to a ~64% drop with 30 mM H_2_O_2_ (Fig. 2B). The results demonstrate that both *Ec*AhpC_1–172_ and *Ec*AhpC_I187G_ are sensitized to inactivation in a magnitude similar to that of *Ec*AhpC_1-186_- YFSKHN, and finally, that the C-terminus, in particular residue I187, is essential for the robustness at high H_2_O_2_ concentrations (Supplementary Fig. S3). The sensitivity of *Ec*AhpC_1–172_ and *Ec*AhpC_I187G_ is surprising, since the destabilized active site, indicated by the increased *Km* value, is supposed to expose the C_P_ for disulphide formation with C_R_’^30^. We propose, that the *Ec*AhpC_1–172,_ and *Ec*AhpC_I187G_ mutants might affect the propensity of the disulphide formation (Supplementary Fig. S3) which would indicate, that the C-terminus plays a role in aiding the disulphide bond formation.

## Discussion

**Figure 1:**
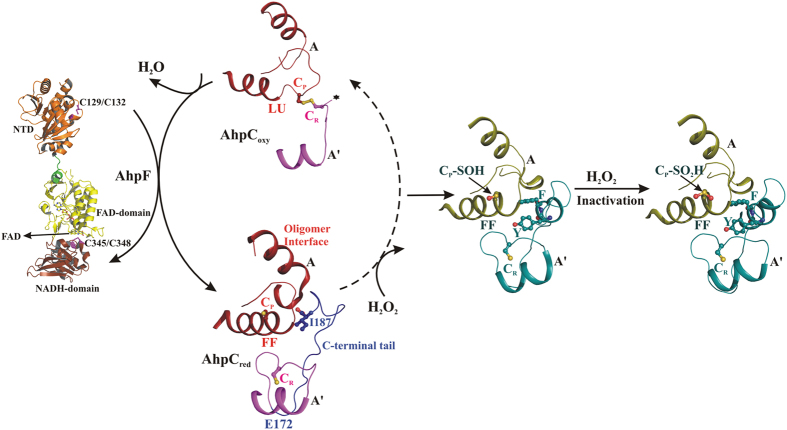
The catalytic cycle of 2-Cys Prxs. The basic functional dimeric unit of 2-Cys Prxs and their active site region is shown. During peroxide reduction, the reduced C_P_ assumes the FF conformation and reacts with H_2_O_2_ to form a C_P_-SOH intermediate. This local unfolding of the active site enables the formation of a disulphide bond with the C_R_ located in the C-terminal tail of adjacent subunit. AhpC is then reduced by AhpF to its FF conformation for future catalytic cycles, with AhpF being oxidized in this process. AhpF is regenerated with the utilization of NADH molecules for further catalytic cycles. In comparison, human Prx is stabilized in the FF active site conformation even after the formation of the intermediate C_P_-SOH form, which eventually promotes over-oxidation.

**Figure 2:**
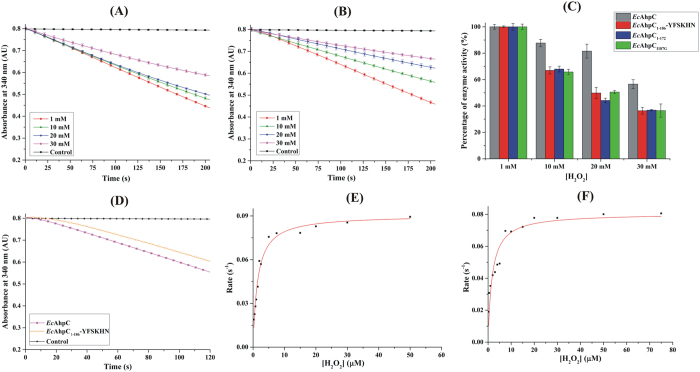
NADPH-dependent peroxidase activity of *Ec*AhpC and *Ec*AhpC_1-186_-YFSKHN. Sensitivity of (**A**) *Ec*AhpC and (**B**) *Ec*AhpC_1-186_-YFSKHN to over-oxidation was determined by the measurement of NADPH oxidation at 1 mM (red), 10 mM (green), 20 mM (blue), and 30 mM H_2_O_2_ (magenta). The background oxidation without Prxs is shown as a control in black. The decrease in NADPH-oxidation with increasing concentrations of H_2_O_2_ is visible for both *Ec*AhpC and *Ec*AhpC_1-186_-YFSKHN. (**C**) Percentage of enzyme activity at each H_2_O_2_ concentration was calculated for WT *Ec*AhpC and its mutants (Mean ± 1 SD) using the rate of NADPH oxidation at each concentration of H_2_O_2_. The rate of activity at the lowest concentration of H_2_O_2_ was taken as 100%. With increasing H_2_O_2_ concentrations, the percentage of enzyme activity decreased. (**D**) NAPDH oxidation of *Ec*AhpC and *Ec*AhpC_1-186_-YFSKHN measured at 30 μM H_2_O_2_ is shown as a representative for all other measurements done at various H_2_O_2_ concentrations to establish the enzyme kinetic parameters. Michaelis-Menten plot of (**E**) *Ec*AhpC and (**F**) *Ec*AhpC_1-186_-YFSKHN was done by fitting data of at least ten concentrations of H_2_O_2_.

Evolutionary adaptation to differing environmental conditions was fundamental to the formation of the different kingdoms of life. Eukaryotic 2-Cys Prxs show a high sensitivity to peroxide inactivation by over-oxidation of the peroxidatic cysteine, C_p_, to sulphinic acid (C_p_-SO_2_H). Due to this adaptation, the enzyme keeps resting levels of H_2_O_2_ low, while permitting higher levels during signal transduction^5^,^18^. The eukaryotic GGLG motif and its extended C-terminal helix, including the YF motif (residues YFSKHN), are responsible for regulating H_2_O_2_ mediated signal transduction^18^. The chimeric *Ec*AhpC_1-186_-YFSKHN described here demonstrates, that transformation of the human C-terminal YFSKHN-helix into the related bacterial AhpC confers sensitivity to over-oxidation on the chimeric enzyme. As for human Prx2 (Fig. 1), the engineered YFSKHN-helix inside the chimeric *Ec*AhpC_1-186_-YFSKHN falls across the active site of the chimeric enzyme, thereby delaying the conformational change from fully folded (FF) to locally unfolded (LU), thereby favoring over-oxidation.

The crystallographic structures of the oxidized and reduced chimeric *Ec*AhpC_1-186_-YFSKHN also provide new insights into the sub-steps during catalysis of peroxide reduction, characterized by the initial formation of a C_P_-SOH intermediate that is generally unstable and prone to further oxidation. This would indicate that the protective mechanism is likely to oppose over-oxidation. The local unfolding (LU) of the active site is such a protection mechanism, but the precise nature of the structural alterations that accompany LU was not known, due to the lack of structural details of this important intermediate C_P_-SOH state in the bacterial 2-Cys Prx family member AhpC. Although the existence of a C_P_-SO_2_H state in the FF active site was demonstrated in human Prx^24^, but did not provide the needed insights for FF to LU transition. As demonstrated by the reduced *Ec*AhpC_1-186_-YFSKHN structure (Fig. 10A-I, -II), a shift of the peroxidatic cysteine, C_P_47, was observed combined with a first turn of the α2-helix, which is now called FF_like_ active site. This novel stage is proposed to mimic the early stage of conformational changes around the active site environment due to the oxidation of C_P_ (Fig. 6A and Supplementary Fig. S4).

The critical step, which decides about inactivation or continues resolving and recycling of AhpC, is combined with the newly described LU_like_ conformation in the reduced *Ec*AhpC_1-186_-YFSKHN structure (Figs 6B and 10A-II). This structurally trapped conformation reveals a severe clash between the active site residues C_P_47 and F45 with the C-terminal tail, causing the C-terminal tail residues L183′, V184′ and I187′, to move away in order to accommodate the preceding structural alteration in the FF active site due to oxidative modification of the peroxidatic cysteine, C_P_. This dynamic behavior of C-terminal tail residues is supported by the FF_like_ structure (Fig. 6A), demonstrating that alterations in the C_P_ and the first turn of the α2-helix caused by oxidation can disrupt interactions of the C-terminal tail, like between L183′, I187′ and the C_P_-loop residues or between I187 with S86A and T88A, the C-terminal tail unfolds partially and moves away from the active site (Fig. 10B). Therefore, alterations of the C-terminal tail confer instability on the FF active site, which in turn increases the rapid shift from the FF to the LU state. These structural rearrangements explain the significant increase of the binding constants observed for the *Ec*AhpC_I187G_ and *Ec*AhpC_S86A,T88A_ mutants (Table 1). Therefore, we propose that the side chain interactions of I187 with the C_P_-loop as well as with amino acids S86A and T88A affect binding of peroxide in the catalytic site.

**Figure 3:**
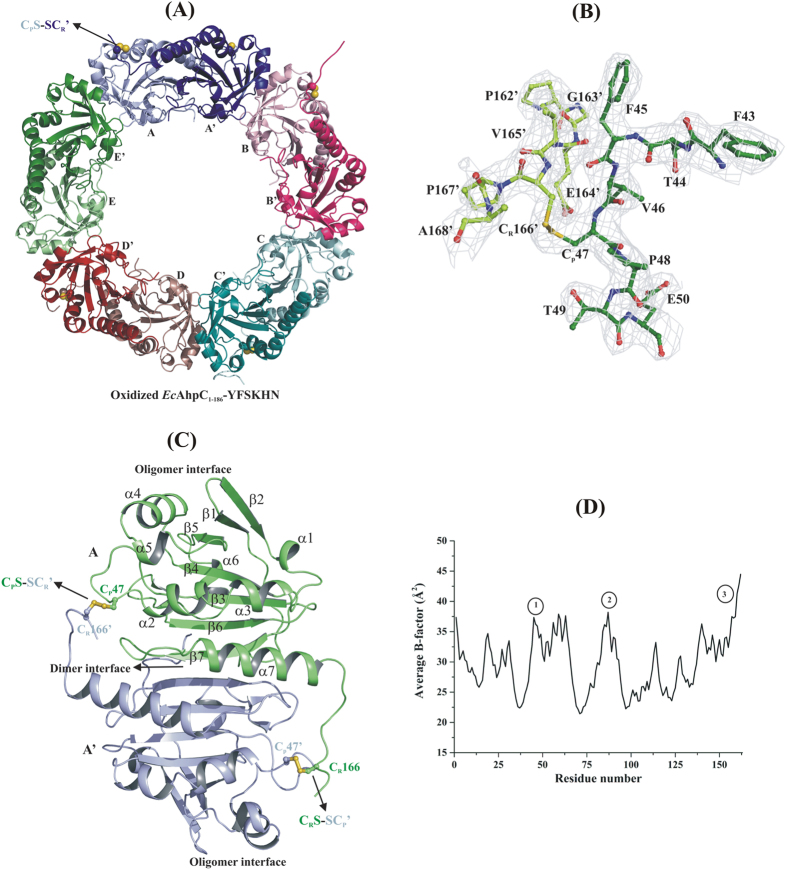
Structural features of oxidized *Ec*AhpC_1-186_-YFSKHN. (**A**) Crystal structure of oxidized *Ec*AhpC_1-186_-YFSKHN in decameric form (α_2_)_5_. Each subunit in the basic functional dimeric unit is shown in light and bright colors denoted as A and A’. The intermolecular disulphide bond between the peroxidatic (C_P_) and resolving (C_R_’) cysteine is shown in ball representation. (**B**) The 2F_O_-F_C_ map contour at 1 σ level around the C_P_ and C_R_’ in disulphide bond conformation. (**C**) Each subunit is composed of two interface regions, namely the dimer and oligomer interface. The secondary structural features are highlighted according to their position in the structure. (**D**) The average main chain B-factor of ten chains showed three highly dynamic regions in the structure.

**Figure 4:**
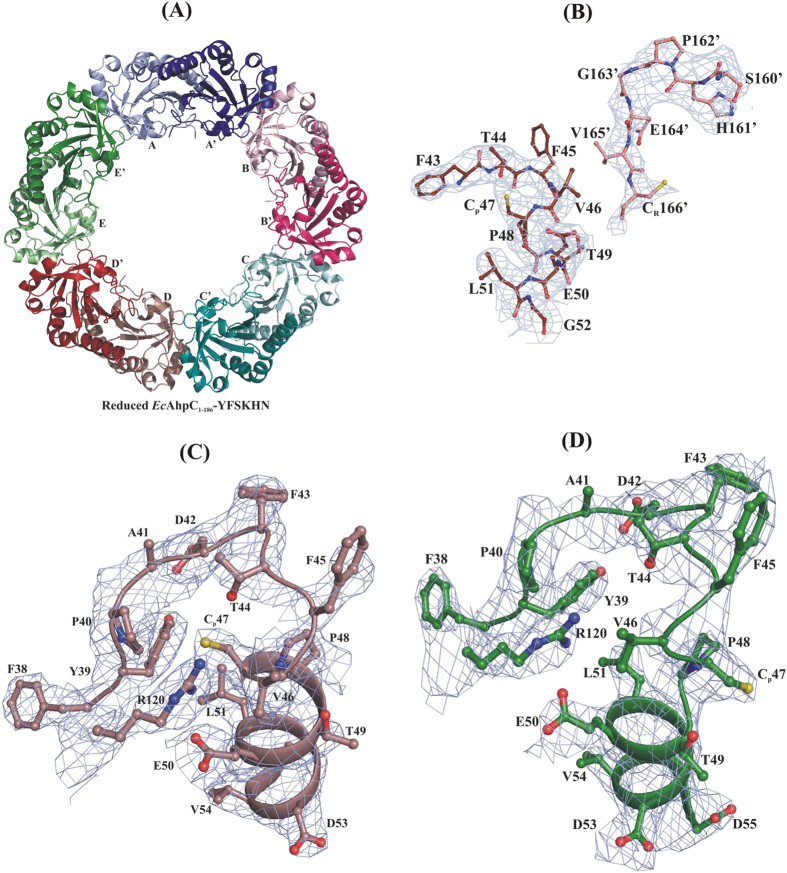
Active site conformation in reduced *Ec*AhpC_1-186_-YFSKHN. (**A**) Decameric structure of the reduced form of *Ec*AhpC_1-186_-YFSKHN in cartoon representation. (**B**) A representative 2F_O_-F_C_ electron density map around the C_P_ and the C_R_ region confirms the reduced state. (**C**) Ribbon representation of the active site, where C_P_47 adopts helical conformation in the reduced *Ec*AhpC_1-186_-YFSKHN structure. The 2F_O_-F_C_ electron density map is shown around the region. (**D**) Ribbon representation with 2F_O_-F_C_ map is shown for the active site region observed once in the *Ec*AhpC_1-186_-YFSKHN reduced structure, wherein C_P_ adopts a loop conformation and is exposed outwards.

**Figure 5:**
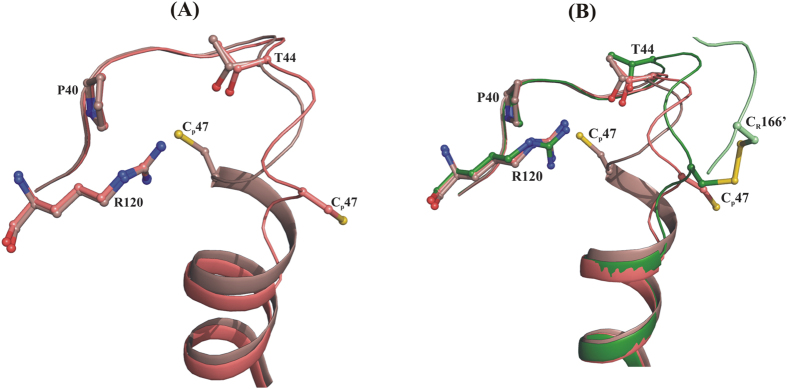
Comparison of FF and LU states from *Ec*AhpC_1-186_-YFSKHN crystal structures. (**A**) Superimposition of two different active sites, the FF_like_- (brown) and LU_like_ state (salmon) which were observed in the reduced *Ec*AhpC_1-186_-YFSKHN structure. (**B**) Comparison of FF_like_- (brown) and LU_like_ state (salmon) in the reduced *Ec*AhpC_1-186_-YFSKHN structure and the LU state of the oxidized *Ec*AhpC_1-186_-YFSKHN (green) structure.

**Figure 6:**
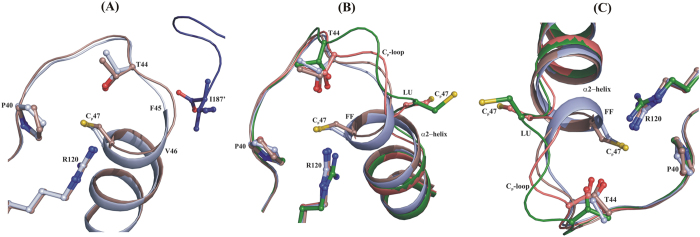
FF to LU transition state like conformation. (**A**) Comparison of the FF_like_ active site of *Ec*AhpC_1-186_-YFSKHN (brown) with the typical FF conformation in the reduced *St*AhpC (light blue), where the C-terminal tail is folded across the active site (blue), which is disordered in reduced *Ec*AhpC_1-186_-YFSKHN. (**B**) The oxidized- (green) and reduced (salmon) active sites observed in the *Ec*AhpC_1-186_-YFSKHN structures are compared with the reduced *St*AhpC (light blue) FF active site. The intermediate FF_like_ (brown) and LU_like_ (salmon) conformations, represent the transition state from the FF to the LU state. (**C**) Another view to close look at the comparison of FF, LU, FF_like_ and LU_like_ active sites.

The comparison of the presented LU_like_ and oxidized structure of *Ec*AhpC_1-186_-YFSKHN also provides novel insights into the resolution of the C_P_-SOH form. As shown in Fig. 10A-III, the transfer from a LU_like_ to a LU state requires the entire C-terminal arm, including residues 166 to 187, to undergo a structural rearrangement that brings the resolving cysteine, C_R_166′, into proximity to finally resolve the C_P_-SOH form. Unfolding of the C-terminal tail might aid disulphide formation by favorably orienting both C_P_ and C_R_’. During the LU conformation, the C_P_ and C_R_’ disulphide bonded active site becomes stabilized (Fig. 10A-IV), which weakens the oligomer building interface^19^. In this oxidized state, the C-terminal tail is highly disordered and not visible in the *Ec*AhpC_1-186_-YFSKHN structure (Fig. 3C), but essential for binding with its reducing partner, the N-terminal domain (NTD) of AhpF (Fig. 10A-V). In this essential regenerative step of the catalytic cycle, the C-terminus of *Ec*AhpC wraps around the NTD and slows the dissociation rate for an efficient electron transfer process^22^. Once the disulphide bond is reduced by the NTD of AhpF, the active site is present in equilibrium between the FF and LU state. In this way, the folding of the C-terminal tail of AhpC back into the active site region and the formation of a stable decamer interface maintain the conformational integrity of the FF active site (Fig. 10A-I). Such a FF active-site pocket is essential to activate and stabilize the C_P_-thiolate for peroxidation^16^.

Establishing the delicate balance between FF and LU conformation and identifying the critical C-terminal tail (amino acids 172 to 187 according to the *Ec*AhpC) and residues (S86, T88) are important as they provide insights not only into the catalytic cycle of Prxs, but also the divergence of the bacterial and human 2-Cys Prxs in robustness and sensitivity to inactivation, respectively. In the sensitive human and more generally eukaryotic Prxs, the two important segments identified here, namely the C-terminal tail and residues S86 and T88, of the less sensitive bacterial Prxs are structurally replaced by the conserved eukaryotic GGLG motif and the YF motif inside the extra C-terminal helix (YFSKHN -helix). Although both the eukaryotic as well as the bacterial enzyme reveal similar catalytic efficiency, the structurally distinct motifs/segments make the eukaryotic enzyme susceptible to over-oxidation^31^. As revealed in Fig. 10A-VI, the eukaryotic C-terminal YFSKHN -helix is complemented by the GGLG motif, forming a stable structural segment across the active site to securely pack the peroxidatic cysteine, C_P_, in the FF conformation, even after the oxidative modification of the C_P_ to the sulfenic acid form^24^,^25^. Based on the proposed model of bacterial AhpC, the C-terminal tail is resistant to unfolding from the active site due to the oxidation induced structural perturbation, thereby delaying the unfolding step needed to switch from the FF active site to the LU conformation. Moreover, the resolution of C_P_-SOH depends on the availability of the resolving cysteine, C_R_’, which is also mediated by the unfolding of the C-terminal tail. This perspective is reflected in the rate of disulphide formation during catalysis, which in case of the bacterial AhpC is significantly higher (75 s^−1^)^32^, when compared to the low rate of 1.7 s^−1^ determined for human Prx2^33^. Taken together, natural selection for the stable C-terminal tail over a stable FF active site reveals the essential role of the C-terminal tail to act as a molecular switch that mediates the structural transition between the FF and LU state during the catalytic cycle.

**Figure 7:**
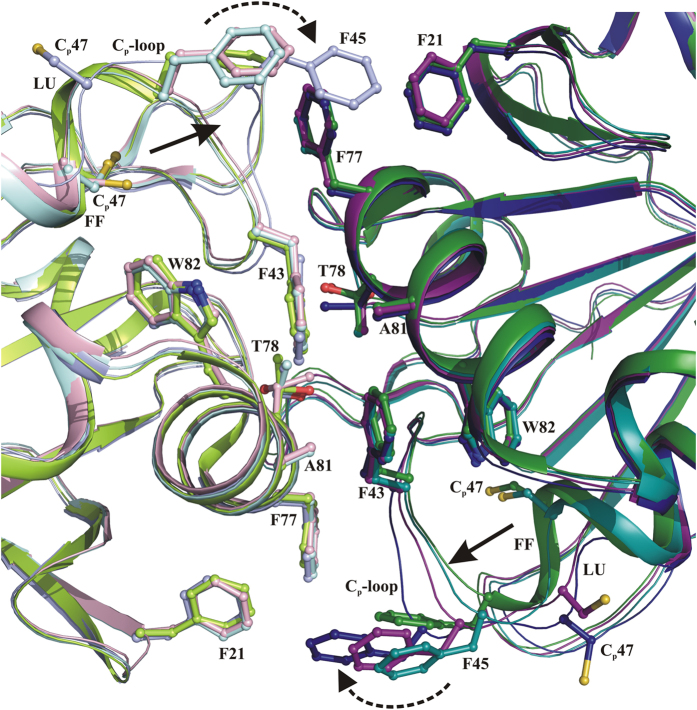
The oligomer interface region. The decamer building interface region between two subunits are shown. The significant structural difference in the interface region is observed only in the residues that form the C_P_-loop. Compared with “FF” (green) C_P_-loop structure, the “FF_like_” (blue), “LU_like_” (magenta) and “LU” (cyan) is shifted away (solid arrow) from the interface region and this facilitates the destabilization of the oligomeric interface through F43 and F45 (dotted arrow). The complementary interface region formed by the neighboring subunit is shown with light colors shade.

**Figure 8:**
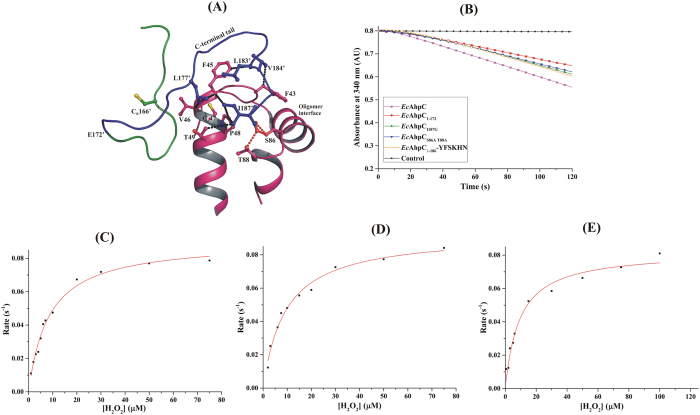
NADPH-dependent peroxidase activity of *Ec*AhpC and C-terminal variants. (**A**) Active site of a typical FF active site of reduced *St*AhpC, where the C-terminal tail folds into the active site and forms weak interactions with the active site residues. The residues S86 and T88 hold the C-terminal tail through hydrogen bond interactions with I187′. (**B**) NADPH oxidation of wild type *Ec*AhpC and its C-terminal variants at 30 μM of H_2_O_2_ is shown as a representative of other measurements done with different concentrations of H_2_O_2_. The background oxidation of *Ec*TrxR and *Ec*Trx without *Ec*AhpC is taken as a control. Michaelis-Menten plots for (**C**) *Ec*AhpC_I187G_, (**D**) *Ec*AhpC_S86A,T88A_, (**E**) *Ec*AhpC_1–172_ were done with various concentrations of H_2_O_2_.

**Figure 9:**
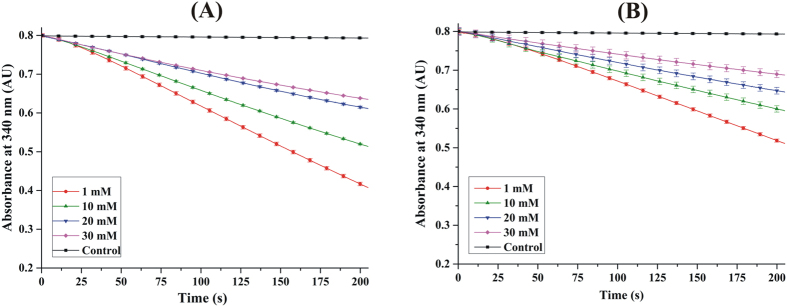
Sensitivity of *Ec*AhpC_I187G_ and *Ec*AhpC_1–172_ to inactivation by H_2_O_2_. Sensitivity of (**A**) *Ec*AhpC_1–172_ and (**B**) *Ec*AhpC_I187G_ to over-oxidation was determined by the measurement of NADPH oxidation at 1 mM (red), 10 mM (green), 20 mM (blue), and 30 mM H_2_O_2_ (magenta). The background oxidation without Prxs is shown as a control in black. The decrease in NADPH-oxidation with increasing concentrations of H_2_O_2_ is visible for both *Ec*AhpC_1–172_ and *Ec*AhpC_I187G_.

**Figure 10:**
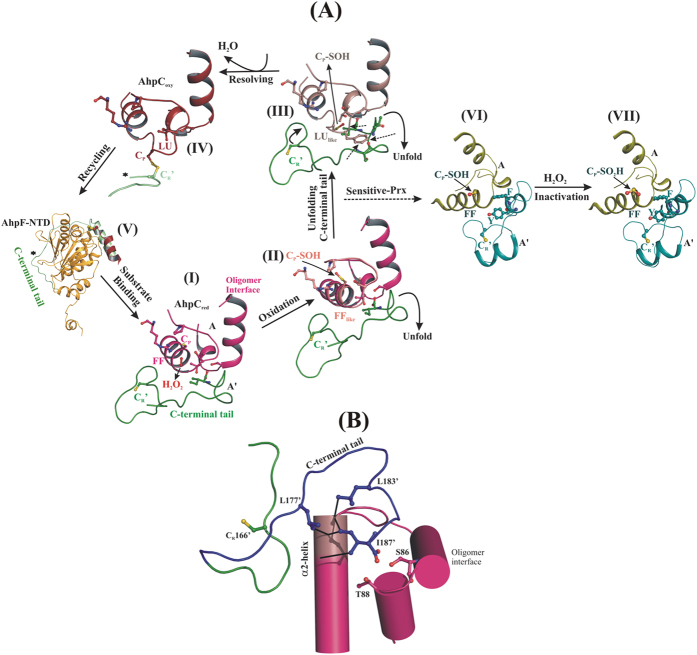
The proposed transition state(s) during the catalytic cycle of Prxs. (**A**) (I) The reduced C_P_ adopts FF conformation and is essential to activate and stabilize the C_P_-thiolate for peroxidation. (II) The oxidative modification of C_P_ to C_P_-SOH leads to localized structural alterations around the conserved active site pocket as seen from the “FF_like_” conformation of the reduced *Ec*AhpC_1-186_-YFSKHN. (III) The “LU_like_” conformation in the reduce *Ec*AhpC_1-186_-YFSKHN structure, reveals a severe clash between the active site residues with the C-terminal tail. The entire C-terminal arm (aa 166–187) is needed to undergo alterations and to enable the disulphide formation between C_R_’ and C_P_ (solid arrow). (IV) The disulphide bonded active site is stabilized in the LU conformation, wherein the C-terminal tail is disordered (denoted by asterisk). (V) The C-terminal tail of AhpC binds to its reducing partner, the N-terminal domain (NTD) of AhpF, to regenerate for another catalytic cycle. (VI) In human Prxs, the extra C-terminal YFSKHN-helix is folded across the active site, thereby delaying the FF to LU transition during the intermediate C_P_-SOH state. In such case, the C_P_-SOH form can further react with peroxide and leads to enzyme inactivation (VII). (**B**) The cartoon representation shows that the C_P_ and the first turn of the α2-helix is stabilized by the weak interactions with the I187 and C-terminal tail, which is hold by residues S86 and T88. Disruption of weak interactions of the C-terminal tail, like between I187 and the α2-helix or between I187 with S86A and T88A, leads to unfolding of the C-terminal tail from the active site region.

The oxidized *Ec*AhpC_1-186_-YFSKHN structure in Fig. 3C,D reveals that the active site loop-helix-motif and the C-terminal tail region adopt structural alteration, bringing C_P_ and C_R_’ to the disulphide bonded LU state. This structural feature is important, since the redox-linked active site conformations are proposed to regulate oligomerization. This regulative mechanism proposes that the disulphide bonded LU state destabilizes the decamer while the C_P_ and C_R_’ reduced FF conformation favors decamer formation^30^. With the studies presented here, we delineate the linkage between the active site conformation and the C-terminal tail residues. Our earlier studies demonstrated that the *Ec*AhpC_1–172_, *Ec*AhpC_1–182_, and *Ec*AhpC_I187G_ mutants prevented decamer formation in solution^21^, leading to the conclusion that the destabilized active site, caused by these mutants, might confer instability to the oligomer building interface (Fig. 7). Taken together, we propose a physical linkage can be established between the three major regions, namely the C_P_ containing active site, the C-terminal tail and the oligomer interface. In the reduced state, this physical linkage enables the folded C-terminal tail and stable oligomer interface to facilitate formation of the FF active site and *vice versa*. However, in the oxidized state, these three structural regions are destabilized^19^,^30^.

In summary, a combination of complementary approaches of genetic engineering, protein chemistry, enzymatic assays, and structural biology has provided new insights into the unique aspect of divergence between the bacterial and human 2-Cys Prxs in robustness and sensitivity to inactivation, respectively. Structural details of the oxidized and reduced chimeric *Ec*AhpC_1-186_-YFSKHN provide novel insights into sub-steps during the catalysis of peroxide reduction, enabling the transition from a fully folded to a locally unfolded conformation. Together with mutational and enzymatic studies these data unravel the fundamental role of the C-terminal tail as a molecular switch that mediates the structural transition between the FF and LU state during the catalytic cycle. Finally, a physical linkage between the C_P_-containing active site, the C-terminal tail and the oligomer interface was established.

## Materials and Methods

### Cloning and overexpression

The chimeric protein *Ec*AhpC_1-186_-YFSKHN, composed of the N-terminal 186 amino acids of *E. coli* AhpC (187 residues) and the C-terminal residues _193_YFSKHN_197_ of human Prx2, was designed based on a protein sequence alignment. *Ec*AhpC_1-186_-YFSKHN was amplified by polymerase chain reaction (PCR) using forward primer 5′-CAT G**CC ATG G**CA ATG TCC TTG ATT AAC ACC AAA ATT AAA CCT-3′ with an *NcoI* restriction site (bold) and reverse primer 5′-GC**G AGC TC**T TAG TTG TGC TTA CTA AAG TAT TTA CCA ACC AGG TCC AG-3′ with a *SacI* restriction site (bold), respectively, and using the pET9-d1-His6 plasmid^34^, which contains the gene encoding *Ec*AhpC^20^. The double mutant, S86 and T88 to alanine (*Ec*AhpC_S86A,T88A_) was amplified using site-directed mutagenesis with Hi-Fi KAPA DNA polymerase (KAPA Biosystems, USA) and the forward primer 5′-AGC AGC GCT GAA GCT ATC GCT AAA ATC AAA TAT GCG-3′ as well as the reverse primer 5′-GAT AGC TTC AGC GCT GCT GTG CCA TGC TTT-3′. The coding sequences of *Ec*AhpC_1-186_-YFSKHN and *Ec*AhpC_S86A,T88A_ were verified by DNA sequencing.

Wild type *E. coli* thioredoxin (Trx) and thioredoxin reductase (TrxR) was amplified by PCR using forward primers 5′-CGT G**CC ATG G**CA ATG AGC GAT AAA ATT ATT CAC CTG-3′ and 5′-TAT G**CC ATG G**GC ACG ACC AAA CAC AGT-3′ with an *NcoI* restriction site (bold) and reverse primers 5′-TA**G AGC TC**G TTA CGC CAG GTT AGC GTC GAG GAA-3′ and 5′-GC**G AGC TC**G TTA TTT TGC GTC AGC TAA AC-3′ with a *SacI* restriction site (bold), respectively. *E. coli* genomic DNA was used as a template.

Liquid cultures were shaken in Luria Broth medium (LB-medium) containing kanamycin (30 μg/ml) at 310 K until an optical density OD_600_ of 0.6–0.7 was reached and isopropyl β-D-1-thiogalactopyranoside (IPTG; final concentration of 1 mM), was added for production of recombinant proteins. The incubation took 3 h at 310 K, except for *Ec*AhpC_S86A,T88A_, which was grown overnight at 291 K after induction. Recombinant *E. coli* AhpC, the C-terminal truncated *Ec*AhpC_1–172_ and mutant *Ec*AhpC_I187G_ were produced by the method described earlier^21^.

### Purification of recombinant proteins

BL21 (DE3) *E. coli* cells containing recombinant *Ec*AhpC, *Ec*AhpC_1–172_, *Ec*AhpC_I187G_, *Ec*AhpC_S86A,T88A_ and *Ec*AhpC_1-186_-YFSKHN were purified according to Dip *et al*.^21^. Recombinant *E. coli* Trx (*Ec*Trx) and **-TrxR were purified with a slightly modified protocol, in which the purified Ni^2+^-NTA fractions were directly loaded on the Superdex 75 HR 10/30- (GE Healthcare) and Superdex 200 column (GE Healthcare), respectively. The purity and homogeneity of the protein samples were analyzed by a 17% SDS-gel^35^. The gels were stained with Coomassie Brilliant Blue G250. The gels of the generated and purified *Ec*AhpC_1-186_-YFSKHN, *Ec*AhpC_S86A,T88A_, *Ec*Trx, and *Ec*TrxR are shown in Supplementary Fig. S5. Protein concentrations were determined using a BioSpec-nano Spectrophotometer (Shimadzu, USA).

### Enzymatic characterization using a peroxidase assay

Peroxide-dependent activity of the various forms of purified recombinant *Ec*AhpC proteins was measured by coupling its activity with NADPH-oxidation (ɛ_280_ = 6220 M^−1^ s^−1^) catalyzed by *Ec*TrxR and *Ec*Trx. The peroxidase activity was carried out at 25 °C by monitoring the decrease in NADPH-absorbance at 340 nm for 120 sec using a stopped-flow spectrophotometer SX20 (Applied Photophysics, UK). The reaction mixture containing 100 μM NADPH, 50 mM HEPES buffer pH 7.0, 100 mM of ammonium sulfate, 0.5 mM EDTA, 0.25 μM of *Ec*TrxR, 4 μM of *Ec*Trx, and 4 μM of *Ec*AhpC were mixed with varying concentrations of hydrogen peroxide (250 nM–100 μM) to initiate NADPH-oxidation. The rate of NADPH-oxidation was calculated by a least square fit to the linear portion of the curve. NADPH consumption measured in the absence of peroxiredoxin was taken as a control. The background rate was subtracted from the experimental rate to determine the activity due to *Ec*AhpC. All rates reported here are the average of three independent experiments.

### Inactivation assay

Peroxide-dependent overoxidation of *Ec*AhpC, -AhpC_1–172_, -AhpC_1187G_ and the chimeric *Ec*AhpC_1-186_-YFSKHN were measured similar to the above mentioned condition, using hydrogen peroxide concentrations of 1–30 mM. NADPH-oxidation was monitored at 340 nm at 25 °C for 210 sec. Background NADPH-oxidation observed for *Ec*Trx and *Ec*TrxR in the absence of Prx, which is significant at higher H_2_O_2_ concentrations, was subtracted to estimate the activity due to Prx. The rate of reaction for each hydrogen peroxide concentration was calculated by fitting the linear portion of the curve. The rate observed for the lowest concentration of hydrogen peroxide is taken as 100% activity, to calculate the remaining enzyme activity in percentage at each concentration of hydrogen peroxide.

### Peroxide reduction assay using SDS-PAGE

This assay is based on the observations that the reduced and oxidized 2-Cys Prxs run as a monomer and dimer, respectively, in a non-reducing SDS-PAGE^33^. Prior to each experiment, WT *Ec*AhpC, *Ec*AhpC_1–172_ and *Ec*AhpC_1187G_ were reduced with 20 mM dithiothreitol (DTT) in 50 mM phosphate buffer, pH 7.4 containing 1 mM diethylenetriaminepentaacetic acid for 1 h. Reduced proteins were separated from excess of DTT using a PD-10 desalting column (GE Healthcare). The different forms of *Ec*AhpC (30 μM) were incubated with varying concentrations of H_2_O_2_ for 5 min. The reaction was stopped by adding 50 mMN‐ethyl maleimide in a sample buffer (4% SDS, 10% glycerol and 62.5 mM Tris–HCl, pH 6.8) and analyzed by a non-reducing 17% SDS-gel.

### Crystallization of *Ec*AhpC_1-186_-YFSKHN

Chimeric *Ec*AhpC_1-186_-YFSKHN was concentrated to 8 mg/ml in buffer containing 50 mM Tris-HCl pH 7.5, 200 mM NaCl, using a Millipore spin concentrator with a molecular-mass cutoff of 10 kDa. An initial crystallization attempt was carried out using the recent protocol of *Ec*AhpC (1.8 M ammonium sulfate, 100 mM MES (2- (*N*-morpholino) ethanesulfonic acid), pH 6.5 and 5% Dioxane)^20^, and the hanging-drop vapour diffusion method in 24-well VDX plates with sealant at 291 K. Diffraction quality crystals were obtained in the optimized condition of 1.6 M ammonium sulfate, 100 mM MES pH 6.5, 5% Dioxane and a protein concentration of 4.5 mg/ml, yielding rod shaped crystals of 0.3 mm × 0.2 mm × 0.1 mm. Reduced *Ec*AhpC_1-186_-YFSKHN crystals were grown by soaking the oxidized crystals with 1 mM Tris(2-carboxyethyl) phosphine (TCEP) for 1–3 min. There was no cracking or disintegration of crystals observed during the soaking.

### Data collection and structure determination

Crystals of *Ec*AhpC_1-186_-YFSKHN were quick-soaked in a cryoprotectant solution containing 25% glycerol in the mother liquid and flash-cooled in liquid nitrogen at 100 K. A single wavelength dataset for both the oxidized and reduced *Ec*AhpC_1-186_-YFSKHN were collected at 140 K, beamline 13B1 of the National Synchrotron Radiation Research Center (NSRRC, Hsinchu, Taiwan) using a ADSC Quantum 315 CCD detector. The diffraction data were indexed, integrated and scaled using Mosflm^36^ and HKL2000 suite^37^. Data collection and processing statistics for oxidized and reduced *Ec*AhpC_1-186_-YFSKHN are summarized in Table 2. Oxidized *Ec*AhpC_1-186_-YFSKHN crystals belong to the monoclinic space group P2_1_ with the unit cell parameters *a* = 99.47 Å, *b* = 134.73 Å, *c* = 107.53 Å and β = 111.06°. The unit cell parameters of reduced *Ec*AhpC_1-186_-YFSKHN are similar to that of the oxidized one (Table 2). The asymmetric unit contains 10 molecules with the solvent content of about 60%. The structure of oxidized *Ec*AhpC_1-186_-YFSKHN was solved using the crystallographic structure of oxidized *Ec*AhpC (PDB ID: 4O5R)^20^ as model for molecular replacement by the program PHASER^38^. The reduced form of *Ec*AhpC_1-186_-YFSKHN was solved using the reduced structure of *Salmonella typhimurium* AhpC (PDB ID: 1N8J)^18^. Refinement^39^ was done until convergence and the geometry of the final model was validated with MolProbity^40^. The figures were generated using PyMOL^41^ and structural comparison analysis was carried out by SUPERPOSE^42^ as included in CCP4 suite^43^.

## Additional Information

****Accession codes:**** The atomic coordinates of the models and their corresponding structure factors of oxidized and reduced EcAhpC1-186-YFSKHN have been deposited in the Protein Data Bank (www.pdb.org) with the entry codes 5B8A and 5B8B, respectively.

**How to cite this article**: Kamariah, N. *et al*. Transition steps in peroxide reduction and a molecular switch for peroxide robustness of prokaryotic peroxiredoxins. *Sci. Rep*. **6**, 37610; doi: 10.1038/srep37610 (2016).

**Publisher's note:** Springer Nature remains neutral with regard to jurisdictional claims in published maps and institutional affiliations.

## Supplementary Material

Supplementary Information

## Figures and Tables

**Table 1:** Kinetic parameters of WT *Ec*AhpC and its mutants.

*Ec*AhpC	*k*_*cat*_ ± SD (s^−1^)	*K*_*m*_ ± SD (μM)	*k*_*cat*/_*K*_*m*_ (M^−1^s^−1^)
WT *Ec*AhpC	0.090 ± 0.001	1.44 ± 0.1	6.2 × 10^4^
*Ec*AhpC_1–172_	0.082 ± 0.004	9.45 ± 0.5	8.6 × 10^3^
*Ec*AhpC_I187G_	0.089 ± 0.003	8.26 ± 0.4	1.0 × 10^4^
*Ec*AhpC_S86A,T88A_	0.089 ± 0.004	9.74 ± 0.5	9.0 × 10^3^
*Ec*AhpC_1-186_-YFSKHN	0.082 ± 0.001	1.77 ± 0.1	4.6 × 10^4^

**Table 2:** Data collection, processing and refinement statistics of *Ec*AhpC_1-186_-YFSKHN.

	Ox. *Ec*AhpC_1-186_-YFSKHN	Red. *Ec*AhpC_1-186_-YFSKHN
Wavelength (Å)	1.000	1.000
Crystal-to-detector distance (mm)	360	420
Rotation range per image (°)	1	1
Total rotation range (°)	140	140
Exposure time per image (s)	5	5
Space group	P2_1_	P2_1_
Unit cell parameters (Å,°)		
*a*=	99.47	100.49
*b*=	134.73	135.65
*c*=	107.53	106.49
α=	90	90
β=	111.06	111.22
γ=	90	90
Molecules in asymmetric unit	10	10
Solvent content (%)	60.72	60.96
Resolution limits (Å)	50.0–2.70 (2.85–2.70)^a^	50.0–3.10 (3.21–3.10)
No. of reflections	202994	138462
Unique reflections	69616	44808
Multiplicity	2.9 (2.9)	3.1 (2.9)
Completeness (%)	96.1 (91.3)	92.9 (92.0)
R_merge_^b^(%)	12.8 (58.4)	11.7 (58.6)
<I//σ(I)>	7.2 (1.8)	8.9 (1.5)
CC_1/2_	98.4 (66.7)	91.5 (66.3)
Refinement statistics		
R-factor^c^ (%)	24.63	25.03
R-free^d^ (%)	28.92	28.36
Number of waters	320	19
Number of sulphates	23	14
Number of glycerol	22	−
MolProbity statistics		
Ramachandran favoured (%)	97.3	98.2
Ramachandran outliers (%)	0	0
Clashscore	0.61	0.39
R.M.S. deviations		
Bond lengths (Å)	0.006	0.007
Bond angles (˚)	0.994	0.946
Overall B values		
From Wilson plot (Å^2^)	48.20	89.60
Mean B value (Å^2^)	30.31	81.83

^a^Values in parentheses refer to the corresponding values of the highest resolution shell.

^b^R_merge_ = ΣΣ_i_|I_h_ − I_hi_|/ΣΣ_i_ I_h_, where I_h_ is the mean intensity for reflection h.

^c^R-factor = Σ||F_O_| − |F_C_||/Σ|F_O_|, where F_O_ and F_C_ are measured and calculated structure factors, respectively.

^d^R-free = Σ||F_O_| − |F_C_|/Σ|F_O_|, calculated from 5% of the reflections selected randomly and omitted during refinement.
