# Supplementary material for: Transition steps in peroxide reduction and a molecular switch for peroxide robustness of prokaryotic peroxiredoxins
Source: Sci Rep. 2016 Nov 28;6:37610. doi: 10.1038/srep37610 (PMC5124861; doi:10.1038/srep37610)
Supplement: Supplementary Information [file srep37610-s1.pdf]

Transition steps in peroxide reduction and a molecular switch for peroxide robustness of prokaryotic peroxiredoxins

Neelagandan Kamariah[a,#], Mun Foong Sek[b,#], Birgit Eisenhaber[a], Frank Eisenhaber[a,c,d] and Gerhard Grüber[a,b,*]

[a] Bioinformatics Institute, Agency for Science, Technology and Research (A*STAR), 30 Biopolis Street, #07-01 Matrix, Singapore 138671, Republic of Singapore

[b] School of Biological Sciences, Nanyang Technological University, 60 Nanyang Drive, Singapore 637551, Republic of Singapore

[c] School of Computer Engineering, Nanyang Technological University (NTU), 50 Nanyang Drive, Singapore 637553, Republic of Singapore

[d] Department of Biological Sciences, National University of Singapore, 8 Medical Drive, Singapore 117597, Republic of Singapore

[#] Authors contributed equally to this work

* To whom correspondence may be addressed:

Prof. Dr. Gerhard Grüber, Tel.: + 65 - 6316 2989; Fax: + 65 - 6791 3856

E-mail: ggrueber@ntu.edu.sg

Keywords: Reactive oxygen species, redox homeostasis, oxidative stress, peroxiredoxins, alkyl hydroperoxide reductase, molecular switch

Supplementary Figure S1. (*A*) Comparison of the ten subunits (chains A-J) of decameric oxidized *Ec*AhpC$_{1-186}$-YFSKHN. Each subunit is shown with different color and the C$_P$47 adopts LU conformation in all subunits. The C-terminal tail is not shown for clarity. (*B*) The dimeric unit (chain A and B: shown in dark and light colors) shows that the C-terminal tail of chain A is stabilized in the oxidized *Ec*AhpC$_{1-186}$-YFSKHN structure by the packing interactions with the symmetry related molecule (grey color). (*C*) The C-terminus of chain H is stabilized by the packing interactions with the symmetry related molecule. Similarly, the C-terminal tail in chain G and J are also stabilized by packing interactions.

Supplementary Figure S2. (*A*) Comparison of the active site conformations between the ten subunits (chains A-J) of decameric reduced *Ec*AhpC$_{1-186}$-YFSKHN. Each subunit are shown with different color and the C-terminal tail is not shown for clarity. The C$_P$47 adopts two different conformation; in one subunit (chain A; red) the C$_P$47 adopts a loop conformation and chains B-J adopt helical conformation. The influence of crystal packing interactions on the active site conformation for (*B*) chain A and (*C*) G is shown as a representative for the rest of the subunits in the decameric reduced *Ec*AhpC$_{1-186}$-YFSKHN. No symmetry related molecule is in contact with the C$_P$47 of the reduced structure.

Supplementary Figure S3. Non-reducing SDS-PAGE analysis of oxidation of (*A*) WT *Ec*AhpC, (*B*) *Ec*AhpC$_{1-172}$ and (*C*) *Ec*AhpC$_{I187G}$ by H_2O_2. The reduced AhpC proteins were treated with varying H_2O_2 concentrations (0 to 30 mM) for 5 min. The SDS-PAGE analysis shows that the reduced AhpC proteins without H_2O_2 run as a major band corresponding to a monomer (*Lane 1*). At 0.1 mM of H_2O_2 *Ec*AhpC becomes oxidized and undergoes the transition from a monomer to

a dimer (*Lane 2*). The monomer band reappears with high concentrations of H_2O_2 (1 to 30 mM) likely due to over-oxidized *Ec*AhpC. The analysis supports that *Ec*AhpC$_{1-172}$ and *Ec*AhpC$_{I187G}$ were sensitive to over-oxidation compared to WT *Ec*AhpC. *D* indicates the dimeric and *M* the monomeric state.

Supplementary Figure S4. Comparison of interaction patterns of the reactive C_P47 with the conserved active site residues between the typical FF (left panel; PDB ID: 4MA9) and FF_{like} (right panel) conformations. A shift in C_P47, located at helix α2 of the FF_{like} active site of reduced *Ec*AhpC$_{1-186}$-YFSKHN, alters its interaction pattern with the conserved active site residues.

Supplementary Figure S5. Gel filtration elution profile of (*A*) *Ec*AhpC$_{1-186}$-YFSKHN, (*B*) *Ec*AhpC$_{S86A,T88A}$, (*C*) *Ec*Trx and (D) *Ec*TrxR. *Ec*AhpC$_{1-186}$-YFSKHN, *Ec*AhpC$_{S86A,T88A}$, and *Ec*Trx were purified by Ni-NTA affinity- and size exclusion chromatography using a Superdex 75 column. In case of *Ec*TrxR, a Superdex 200 gel filtration column was used. The volume of the shaded peak area was pooled and 3 µl of pure eluted protein were applied on a 17% SDS gel to estimate its purity (*inset*).

(A)

(B) (C)

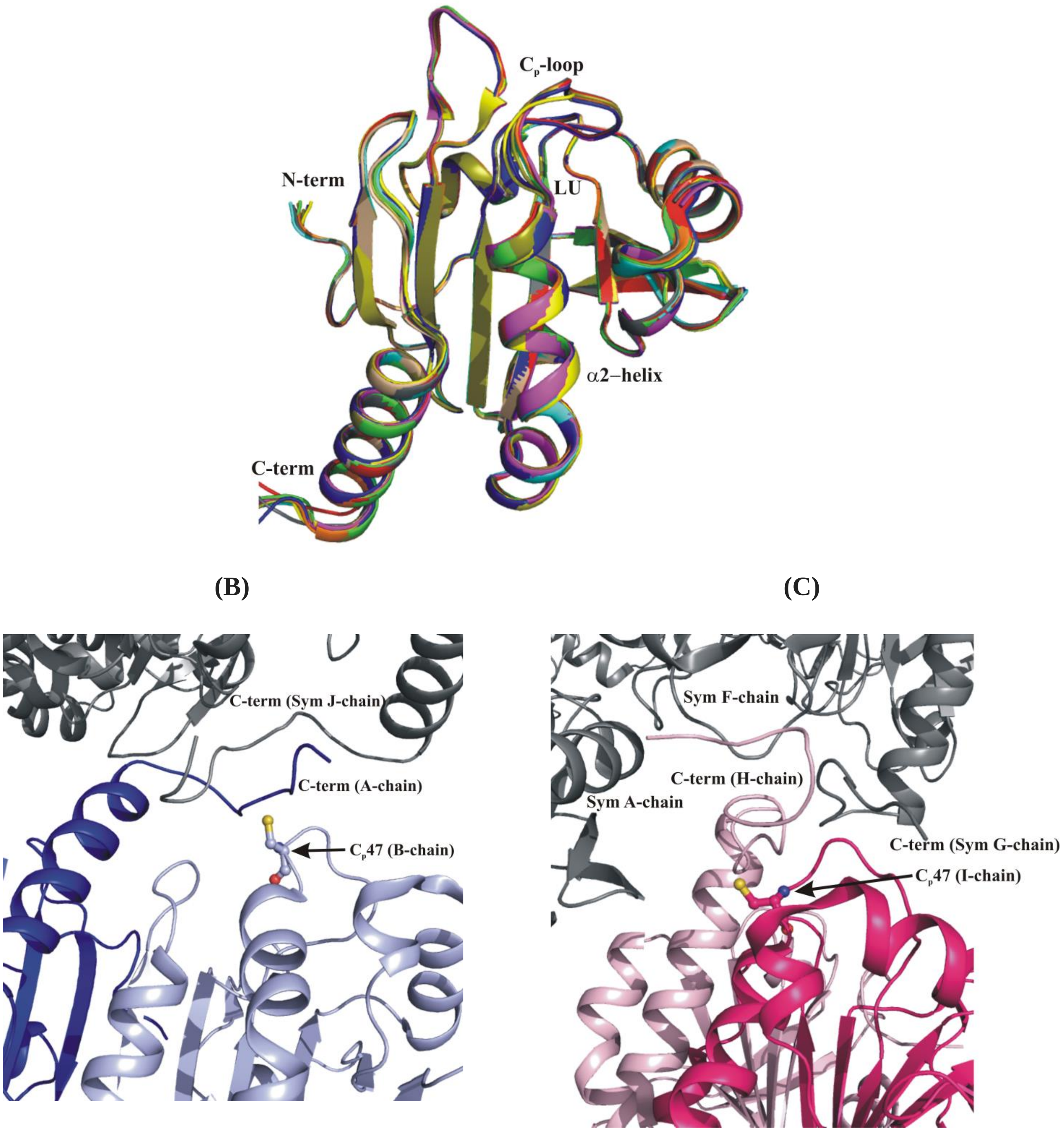

Supplementary Figure S1A-C

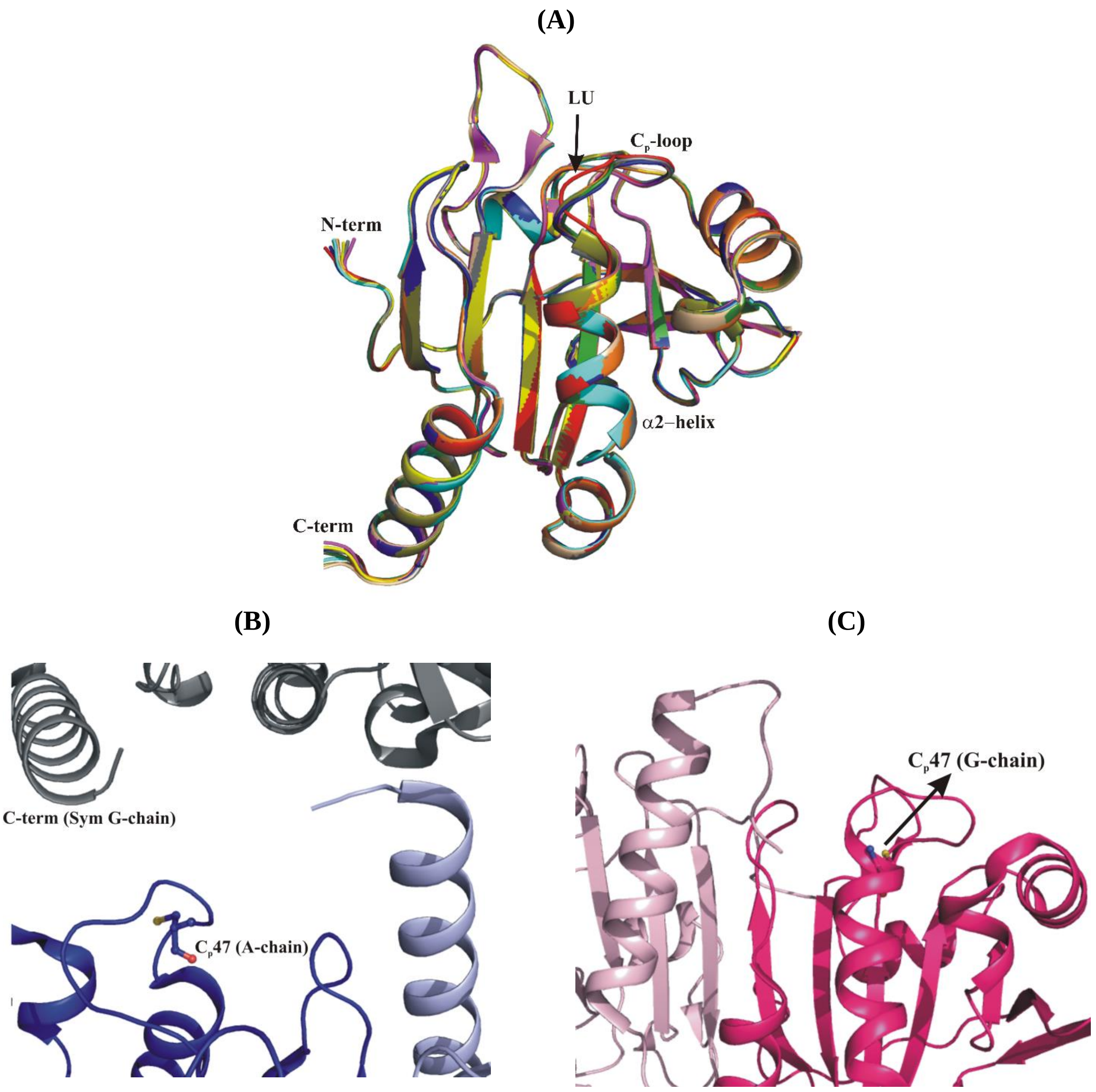

Supplementary Figure S2A-C

(A)

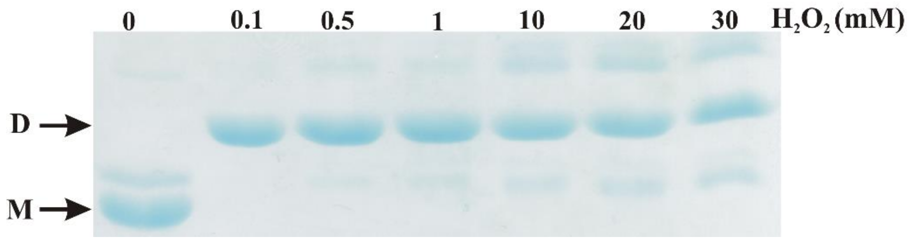

(B)

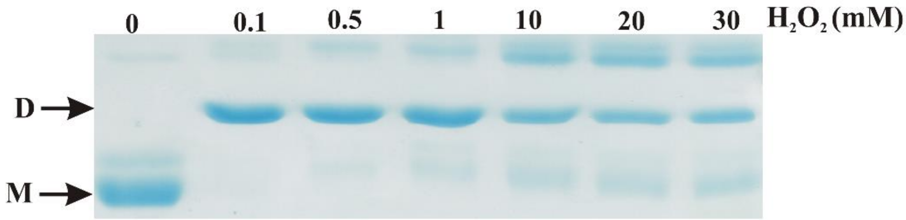

(C)

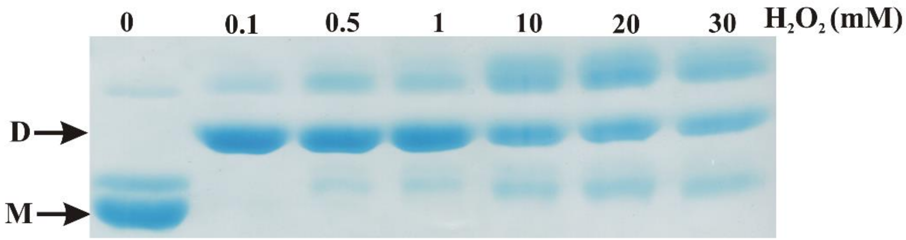

Supplementary Figure S3A-C

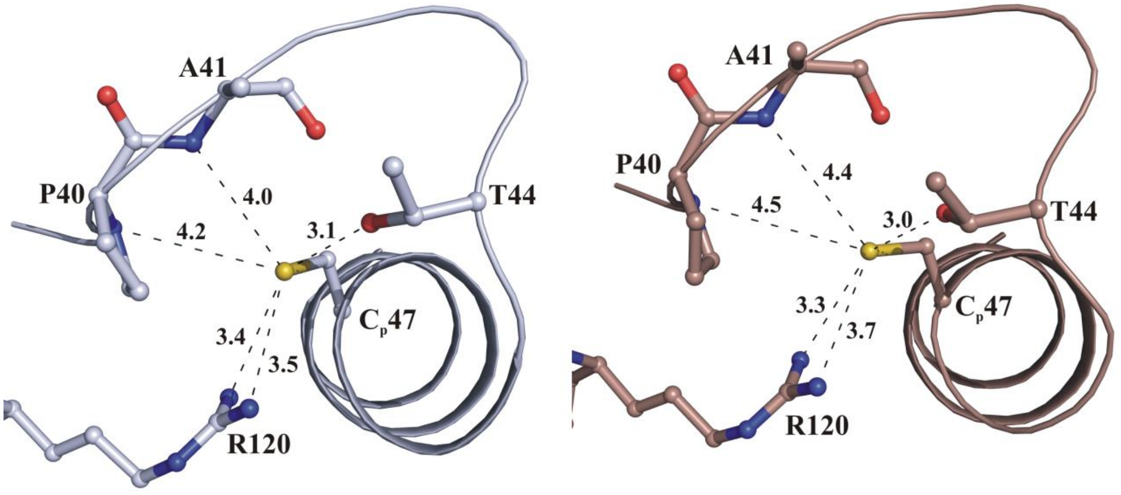

Supplementary Figure S4

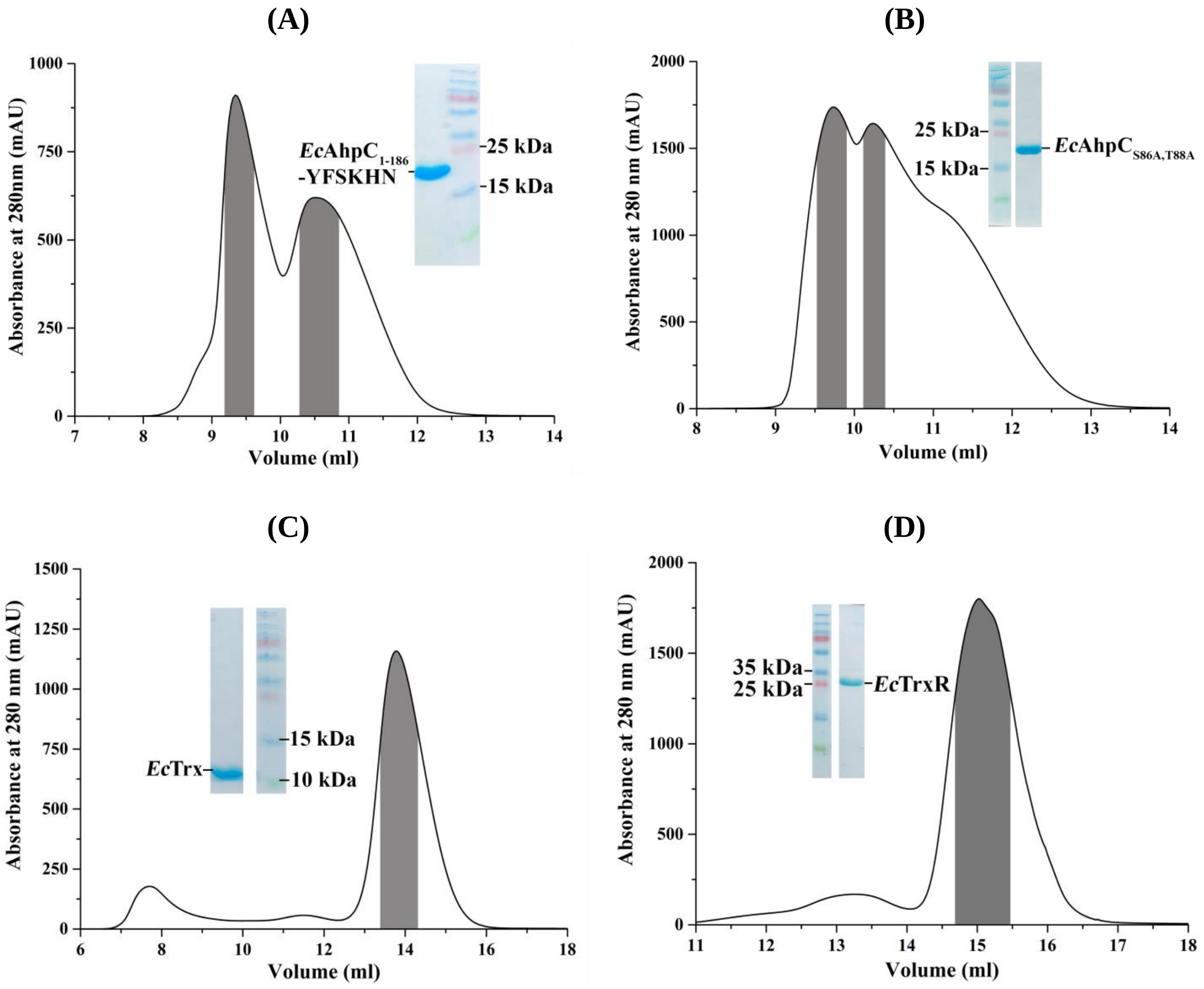

Supplementary Figure S5A-D

Supplementary Table S1: The backbone dihedral angles for active site residues at different conformations

Conformation	Residue	Phi (°)	Psi (°)
	P48	-60.6	-36.5
FF	C47	-49.2	-53.6
	V46	-71.8	-35.4
	F45	115.8	-61.7
	P48	-88.6	48.5
LU	C47	-55.5	132.7
	V46	-58.1	132.6
	F45	-79.2	-21.3
	P48	-61.0	-34.5
FF_{like}	C47	-64.3	-38.9
	V46	-85.9	-30.8
	F45	-86.7	-35.6
	P48	-65.5	52.0
LU_{like}	C47	-53.1	150.4
	V46	-67.0	90.1
	F45	-99.9	-15.3
